# Explaining Associations Between Adverse Childhood Experiences and Spiritual Well‐Being Through Resilient Mindset, Depression, Anxiety, and Stress Among Turkish University Students

**DOI:** 10.1002/brb3.71227

**Published:** 2026-02-10

**Authors:** Gülçin Güler Öztekin, Nouf Abdullah Alshehri, Abdulmohsen Mohammed Abdullah Alkhulayfi, Murat Yıldırım

**Affiliations:** ^1^ Department of Psychology, Faculty of Science and Letters Agri Ibrahim Cecen University Ağrı Türkiye; ^2^ Department of Education and Psychology University of Hafr Al Batin Saudi Arabia; ^3^ Department of Business Administration, Faculty of Economics and Administration King Abdulaziz University Jeddah Saudi Arabia; ^4^ Psychology Research Center Khazar University Baku Azerbaijan

**Keywords:** adverse childhood experiences, anxiety, depression, resilient mindset, spiritual well‐being, stress

## Abstract

**Purpose:**

This study addresses an important gap in understanding the mechanisms linking adverse childhood experiences (ACEs) to spiritual well‐being. Specifically, it tested the serial mediating roles of resilient mindset and psychological distress—depression, anxiety, and stress—in both independent and sequential pathways. By controlling for age and gender, the study aimed to clarify how resilience and distress jointly explain the relationship between ACEs and spiritual well‐being.

**Method:**

A cross‐sectional design was used to conduct this study. A total of 686 university students participated in this study (75.4% females; *M  =*  21.5, SD  =  2.21).

**Finding:**

The results showed that higher ACEs were associated with lower spiritual well‐being, with a significant total effect (*B =* −0.64). Significant indirect effects supported the mediating roles of resilient mindset (*B =* −0.06) and psychological distress, including depression (*B =* −0.14), anxiety (*B =* −0.09), and stress (*B =* −0.09). Serial mediation analyses further showed that ACEs were indirectly associated with spiritual well‐being through resilient mindset, followed by depression (*B =* −0.03), anxiety (*B =* −0.01), and stress (*B =* −0.01).

**Conclusion:**

These findings suggest that developing a resilient mindset and reducing psychological distress may be important in reducing the effects of ACEs on well‐being.

## Graphical abstract

This study investigates how adverse childhood experiences (ACEs) influence spiritual well‐being among Turkish university students. Findings reveal that higher ACEs are linked to lower spiritual well‐being. Resilient mindset and psychological distress (depression, anxiety, and stress) act as independent and sequential mediators in this relationship. Specifically, ACEs reduce resilient mindsets, which in turn increases depression, anxiety, and stress, ultimately lowering spiritual well‐being. Strengthening resilience and alleviating psychological distress may help buffer the negative impact of ACEs on well‐being.

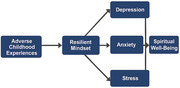

## 1 Introduction

Spirituality is essential to the identity and existence of human beings and to coping with adverse life circumstances (Chirico et al. 2023; Makkaoui et al. 2024; Nahardani et al. 2019). Spirituality is a dynamic and functional concept and is considered personal and relational (Zinnbauer et al. 1999). Spiritual well‐being refers to a state of being that reflects positive emotions, behaviors, and cognitions of one's relationships with the transcendent, oneself, nature, and others. This state provides the individual with identity, integrity, inner harmony, fulfillment, beauty, joy, satisfaction, respect, love, positive attitudes, and a sense of purpose (Gomez and Fisher 2003). Higher spiritual well‐being has been associated with greater life satisfaction and higher hope (Özdemir et al. 2023). Spiritual well‐being is a key constituent of health‐related quality of life (Bredle et al. 2011). This well‐being is a protective factor in psychological, mental, and physical health (Coppola et al. 2021). In addition, individual, behavioral, or social factors such as hope, religious practice, and social support are predictors of spiritual well‐being (Chaiyasit et al. 2020). Childhood experiences are another factor affecting spiritual well‐being (Maral et al. 2024).

ACEs are related to traumatic events, which can have lasting deleterious effects on individuals’ health and well‐being. These experiences include maltreatment, sexual and emotional abuse, and living in an environment that is detrimental to children's developmental process (Boullier and Blair 2018). The events that will cause psychological distress among children, such as growing up in a home where there is domestic violence, substance abuse in the home, or divorce of parents, are some of the adverse experiences of children during childhood (Gündüz et al. 2018). These experiences may lead to short‐term, medium‐term, and long‐term adverse consequences for health and development, and their effects can be exponential, particularly during critical periods of vulnerability and developmental plasticity (Bhutta et al. 2023). These negativities may form the basis of problems such as physical and psychological distress, risky behaviors, and developmental disorders (Kalmakis and Chandler 2015). It is also possible for individuals to experience significant distress or conflict in spirituality after experiencing trauma or adversity in childhood (McCormick et al. 2017). Therefore, identifying this relationship and the factors involved in this relationship can contribute to the successful progress of development.

### 1.1 ACEs and Spiritual Well‐Being

Family experiences play a crucial role in the formation of spiritual well‐being. In particular, an individual's family history and childhood experiences significantly affect later spiritual experiences and satisfaction. Maltreatment and negative experiences during this period damage an individual's value system, which can negatively affect spiritual well‐being (Arslan 2025). Previous literature has provided evidence of the impact of ACEs on spiritual well‐being. For example, childhood traumas such as emotional, sexual, and physical abuse and neglect were associated with an increased level of spiritual struggles (Janů et al. 2022). Individuals who experience adversities like abuse and maltreatment early in life have difficulties in emotion regulation and intolerance of uncertainty. This causes a decrease in the level of spiritual well‐being in individuals (Yilmaz and Satici 2024). Based on the above empirical data, the first hypothesis was formed as follows.

H1: ACEs have a direct effect on spiritual well‐being.

### 1.2 Resilient Mindset as a Mediator

“Resilient mindset” refers to being aware of the psychological, physical, spiritual, and social resources to deal effectively with a specific situation. Based on this definition, it can be asserted that establishing resilience is based on developing a mindset that allows individuals to overcome or adapt to these challenges in a positive way (Arslan and Wong 2024). Today, resilience is not only the ability to survive adversity but also a dynamic, flexible, and multidimensional structure that emphasizes the building of a resilient mindset (Wong 2020). Developing a resilient mindset is an essential resource for handling life's challenges. The effects of the adversities experienced by human beings in childhood continue to haunt the individual in later periods, which restricts their development (Hoskeri 2024). It may not be possible to achieve the well‐being required for the maintenance of a quality of life in individuals who cannot form a resilient mindset (Hansen et al. 2021). Resilient individuals can deal with stress and adapt to change. In this way, they can boost their functioning and well‐being despite adversity. In addition, in the previous literature, there is a study confirming the mediating role of resilient mindset in the link between adverse life circumstances and well‐being. In that study, resilient mindset buffered the adverse impact of stress related to the coronavirus on depressive symptoms (Arslan and Coşkun 2024).

A resilient mindset is a cognitive‐motivational resource that enables individuals to view stressful and negative life experiences as manageable and meaningful. This assessment approach can strengthen the fundamental components of spiritual well‐being—meaning, purpose, and harmony—by placing challenging experiences within a broader life story context, preserving a sense of purpose and consistency, and activating meaning‐based coping processes in the face of existential threats (Park 2010; Wong 2013). In this regard, a resilient mindset serves as a functional bridge between cognitive coping assessments and the existential and transcendent dimensions of spiritual well‐being (Pargament 2011). Thus, the second hypothesis was formed as follows:

H2: Resilient mindset has a mediating role in the link between ACEs and spiritual well‐being.

### 1.3 Depression, Anxiety, Stress, and Resilient Mindset as Mediators

Depression refers to symptoms of decreased mood, physical retardation, and rumination, and major life transitions may lead to depression if they are major, sudden, and result in the loss or change of life roles (Moustafa et al. 2020). Anxiety is a psychological condition that occurs when a person feels uneasiness, anxiety, and tension in the face of uncertain situations (Zeidner and Matthews 2010). Stress is caused by cognitive or emotional factors, including worries or perceived threats (Liu et al. 2021; Roussis and Wells 2008). Stress is a state that occurs when an individual perceives environmental demands beyond his or her assessed capacity (Vingerhoets 2008). Cognitive factors contribute to preventing the emergence of psychological distress (Öztekin et al. 2025). Traumatic events and adverse experiences overwhelm an individual's ability to cope and can lead to symptoms such as intrusive memories, avoidance behaviors, and hyperarousal (Fossion et al. 2015). Adverse experiences in childhood have a relationship with the development of poor health consequences.  More importantly, individuals with more ACEs had more mental health problems (Hedrick et al. 2021). Depression, anxiety, and stress were also associated with lower spiritual well‐being (Manyema et al. 2018). In other words, the probability of experiencing high spiritual well‐being was lower when experiencing psychological distress (Fabbris et al. 2017). In addition, a growing literature provides evidence for the mediating role of psychological distress. For example, parental ACEs increased the levels of psychological distress, which led to more frequent use of harsh discipline and ultimately increased children's problematic media use (Zhu et al. 2024). Psychological distress mediated the association between ACEs and addictions such as smoking (Strine et al. 2012) and alcohol use (Shin et al. 2015). Psychological vulnerability negatively impacted individuals' well‐being through psychological distress (Hatun 2024).

Mental health problems such as stress, depression, and anxiety are highly linked to psychological variables. Coping strategies influence anxiety among physicians (Rizzo et al. 2023), while perceptions of severity and controllability predict stress in healthcare workers (Yıldırım and Özaslan 2022). Psychological capital protects adolescents against internalizing problems and enhances well‐being (Yıldırım et al. 2025). Psychological flexibility reduces posttraumatic stress disorder and adjustment difficulties (Yıldırım et al. 2024a), and occupational stress consistently predicts higher anxiety and depression (Yıldırım et al. 2024b). Therefore, we assumed that depression, anxiety, and stress would have a disruptive effect on the relationship to spiritual well‐being in individuals who had ACEs and formulated the third hypothesis as follows.

H3: Depression, anxiety, and stress have a mediating role in the link between ACEs and spiritual well‐being.

From the perspective of developmental psychopathology, mental health problems lead to deviations from a healthy developmental process over time (Sroufe 1997). Transformations that occur as part of normative development are assessed to help us understand the problem. The effects of the problem can vary significantly depending on the developmental stage. ACEs can be considered as one source of these problems. It is essential to comprehend how ACEs affect children's mental health and to consider the timing of such experiences (Hawes and Allen 2023). Developmental psychopathologists are interested in investigating the interrelationships among dynamic systems and the processes that characterize system disruption and in elucidating the mechanisms by which compensatory, self‐correcting tendencies are initiated when higher‐level observers detect deviations in a self‐contained system within a larger system (Cicchetti and Rizley 1981). Accordingly, developmental psychopathologists argue that examining the mechanisms and factors that enhance positive adjustment among individuals experiencing significant adversity is as important as investigating developmental trajectories toward maladjustment and psychopathology. According to this theory, risk and protective factors must be considered together, and one protective factor is the development of a resilient mindset, which is a dynamic developmental process that consists of the achievement of positive adaptation despite exposure to trauma, severe adversity, or significant threat, which typically constitutes major insults to the processes underlying biological and psychological development (Cicchetti 2013). Accordingly, we can assume that a resilient mindset will protect against ACEs that lead to psychological problems and impair spiritual well‐being.

Previous literature separately presents evidence for the negative relationship between ACEs and resilient mindset and spiritual well‐being (Janů et al. 2022), the positive relationship between ACEs and psychological distress (Hedrick et al. 2021), the negative relationship between psychological distress and spiritual well‐being (Fabbris et al. 2017), and the negative and positive relationships between resilient mindset and psychological distress and well‐being, respectively (Arslan and Coşkun 2024). In addition, only one study has examined the protective role of the resilient mindset in the association between stress and depressive symptoms (Arslan and Coşkun 2024), which we have noted its importance from a developmental psychopathology perspective. However, there is no research examining the mediating effects of resilient mindset and psychological distress on the relationship between ACEs and spiritual well‐being. This study positions spiritual well‐being not as a passive consequence of ACEs, but as a dynamic construct that mediates the meaning‐making of early experiences. Furthermore, testing resilient mindset as a cognitive mechanism that protects spiritual well‐being and psychological distress as a process that weakens this relationship within the same mediating model offers a unique integrated approach, limited in the existing literature. Therefore, we generated the following hypotheses.

H4: Resilient mindset and depression have a chain mediating role in the association between ACEs and spiritual well‐being.
H5: Resilient mindset and anxiety have a chain mediating role in the association between ACEs and spiritual well‐being.
H6: Resilient mindset and stress have a chain mediating role in the association between ACEs and spiritual well‐being.

## 2 Method

### 2.1 Participants

A total of 686 Turkish university students participated in the study, comprising 517 females (75.4%) and 169 males (24.6%). The students’ ages ranged from 18 to 32 years. The mean age was 21.5  years (SD =  2.21). Most of the participants were juniors (n = 200), followed by freshmen (*n =* 170), sophomores (*n =* 155), seniors (*n =* 116), preparatory students (*n =* 30), and fifth‐year students (*n =* 15). 102 students perceived their childhood as very good; 401 students perceived it as good; 117 students were neutral; 56 students perceived it as bad; and 10 students perceived it as very bad.

### 2.2 Measures

#### 2.2.1 Adverse Childhood Experiences Scale

This scale was developed by CDC and Permanente (1997) and adapted to Turkish culture by Gündüz et al. (2018) to question the presence of adverse experiences such as sexual violence, physical violence, emotional violence, abuse, and physical and emotional neglect in childhood. The scale consists of 10 items with a yes‐no response (e.g., “Did a parent or other adult in the household …often or very often swear at, insult, or put you down? Often or very often act in a way that made you afraid that you would be physically hurt?”). The total score of the scale varies between 0 and 10. Zero points indicate no exposure to ACEs. Cronbach's alpha value was 0.74. In this study, Cronbach's alpha value was 0.75.

#### 2.2.2 Resilient Mindset Scale

The scale was developed by Arslan and Wong (2024) to assess the attitudes and beliefs that contribute to an individual's ability to cope with challenges. The scale consists of 6 items on a 5‐point Likert‐type scale, scored from 0 (almost never true) to 4 (almost always true) (e.g., “I have the mental and emotional toughness to withstand the challenges that I face or come my way”). The total score of the scale varies between 0 and 24. Higher scores indicate higher resilient mindset levels. Cronbach's alpha value was 0.86. In this study, Cronbach's alpha value was 0.84.

#### 2.2.3 Depression, Anxiety and Stress Scale

The 42‐item Depression, Anxiety and Stress Scale, developed by Lovibond and Lovibond (1995) to measure people's psychological states, has three subscales (depression, anxiety, and stress). This scale was shortened by Henry and Crawford (2005), and the adaptation of this version to Turkish culture was done by Yılmaz et al. (2017). The scale consists of 21 items on a 4‐point Likert‐type scale, scored from 0 (not suitable for me) to 3 (completely suitable for me) (e.g., “I realized I couldn't have any positive emotions”). The total score of each subscale varies between 0 and 21. Higher scores indicate higher depression, anxiety, and stress levels. Cronbach's alpha value was 0.81 for depression, 0.80 for anxiety and 0.75 for stress subscales. In this study, Cronbach's alpha value was 0.81, 0.82, and 0.84, respectively.

#### 2.2.4 Spiritual Well‐Being Scale

The scale was developed by Bredle et al. (2011) and adapted to Turkish culture by Arslan and Yıldırım (2021) to assess the spiritual well‐being of people. The scale consists of 5 items on a 5‐point Likert‐type scale, scored from 0 (not at all) to 4 (very much) (e.g., “I feel a sense of purpose in my life”). The total score of the scale varies between 0 and 20. Higher scores indicate higher spiritual well‐being levels. Cronbach's alpha value was 0.85. In this study, Cronbach's alpha value was 0.72. McDonald's omega indicated acceptable internal consistency (ω = 0.77).

### 2.3 Procedure

The Agri Ibrahim Cecen University Ethics Committee confirmed this research (Ethics Code: E‐95531838‐050.99‐124706). The convenience sampling method was employed to recruit participants for the study. Data was collected online via Google Forms. Detailed information about the study was provided in the survey preface. Consent was obtained from each participant before completing the survey.

### 2.4 Data Analysis

A priori power analysis using G*Power indicated that a sample size of 138 would provide 95% power to detect a medium effect size (*f* ^2^ = 0.15) at *α* = 0.05. Given the current sample size (*N =* 686), the study had sufficient power (1.00) to detect a medium effect size (*f* ^2^ = 0.15) at *α* = 0.05, indicating that the sample was adequate for the planned analyses.

Skewness and kurtosis values were identified to test the assumption of normality with scores less than |2| (Curran et al. 1996). ACEs scores ranged from 0 to 10 (median = 0, 25th–75th percentile = 0–2) and were treated as a continuous variable for analyses. The relationships between study variables were examined with Pearson correlation analysis. The basic assumptions were checked before the mediation analysis. Regarding the assumptions of linearity, homoscedasticity, independence, and multicollinearity, no issues were preventing the running of mediation analysis (tolerance ≥ 0.45 and VIF ≤ 2.20) (Kutner et al. 2004). To test the proposed mediation model, we used PROCESS‐Macro v4.2 with model 81. The results were evaluated with standardized and unstandardized regression coefficients and squared multiple correlation values. We examined the indirect effects of mediators at 95% confidence intervals using a bootstrapping technique with 5.000 bootstrap samples. All analyses were performed using SPSS version 27.

## 3 Results

Descriptive analysis showed that skewness and kurtosis values indicated a normal distribution (see Table 1). The results of the correlation analysis revealed that ACEs had significant positive correlations with depression, anxiety and stress, while significant negative correlations with resilient mindset and spiritual well‐being. Resilient mindset had a significant positive correlation with spiritual well‐being, while depression, anxiety, and stress had a significant negative correlation with spiritual well‐being. These results are presented in Table 1.

**TABLE 1:** Descriptive statistics, skewness, kurtosis, and correlations.

Variables	M	SD	Skewness	Kurtosis	Correlation					
					1	2	3	4	5	6
1. ACEs	1.17	1.75	1.56	1.56	—					
2. Resilient mindset	14.31	4.64	−0.20	−0.18	−0.17**	—				
3. Depression	6.39	3.42	0.26	−0.09	0.40**	−0.41**	—			
4. Anxiety	5.79	3.58	0.28	−0.33	0.39**	−0.25**	0.53**	—		
5. Stress	6.57	3.82	0.17	−0.31	0.36**	−0.25**	0.54**	0.70**	—	
6. Spiritual well‐being	13.14	3.41	−0.44	−0.01	−0.33**	0.36**	−0.48**	−0.43**	−0.44**	—

Abbreviations: SD = standard deviations; M = mean,

**p < 0.001.

**FIGURE 1:**
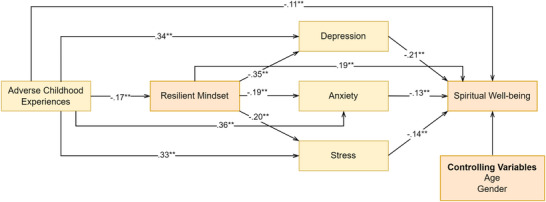
The mediation model with standardized coefficients.

After controlling for age and gender, the results of the mediation analysis revealed that ACEs were significantly associated with resilient mindset (*B =* −0.46, *p <* 0.001), depression (*B =* 0.66, *p <* 0.001), anxiety (*B =* 0.74, *p <* 0.001), and stress (*B =* 0.72, *p <* 0.001). Resilient mindset was significantly associated with depression (*B =* −0.26, *p <* 0.001), anxiety (*B =* −0.15, *p <* 0.001), and stress (*B =* −0.16, *p <* 0.001). ACEs (*B =* −0.21, *p <* 0.001), resilient mindset (*B =* 0.14, *p <* 0.001), depression (*B =* −0.21, *p <* 0.001), anxiety (*B =* −0.12, *p <* 0.001), and stress (*B =* −0.12, *p <* 0.001) were significantly associated with spiritual well‐being. They explained 33% of the variance in spiritual well‐being (see Table 2). The standardized indirect effects for the mediation model are illustrated in Figure 1.

**TABLE 2:** Unstandardized coefficients for the mediation model.

Predictor	Outcome	Coeff.	SE	*t*	*p*	
ACEs	Resilient mindset	−0.46	0.10	−4.58	0.00	*R* ^2^ = 0.03 *F =* 8.26; *p <* 0.001
Gender	Resilient mindset	−0.38	0.41	−0.91	0.36	
Age	Resilient mindset	0.13	0.08	1.65	0.09	
ACEs	Depression	0.66	0.06	10.28	0.00	*R* ^2^ = 0.29 *F =* 68.65; *p <* 0.001
Resilient mindset	Depression	−0.26	0.02	−10.83	0.00	
Gender	Depression	0.14	0.26	0.56	0.57	
Age	Depression	0.01	0.05	0.23	0.81	
ACEs	Anxiety	0.74	0.07	10.44	0.00	*R* ^2^ = 0.20 *F =* 41.88; *p <* 0.001
Resilient mindset	Anxiety	−0.15	0.02	−5.56	0.00	
Gender	Anxiety	−0.85	0.29	−2.92	0.00	
Age	Anxiety	0.02	0.05	0.36	0.71	
ACEs	Stress	0.72	0.07	9.29	0.00	*R* ^2^ = 0.17 *F =* 36.16; *p <* 0.001
Resilient mindset	Stress	−0.16	0.02	−5.68	0.00	
Gender	Stress	−0.47	0.31	−1.48	0.13	
Age	Stress	0.12	0.06	2.01	0.04	
ACEs	Spiritual well‐being	−0.21	0.06	−3.10	0.00	*R* ^2^ = 0.33 *F =* 48.78; *p <* 0.001
Resilient mindset	Spiritual well‐being	0.14	0.02	5.52	0.00	
Depression	Spiritual well‐being	−0.21	0.04	−5.13	0.00	
Anxiety	Spiritual well‐being	−0.12	0.04	−2.81	0.00	
Stress	Spiritual well‐being	−0.12	0.04	−3.01	0.00	
Gender	Spiritual well‐being	−0.05	0.25	−.22	0.82	
Age	Spiritual well‐being	−0.11	0.05	−2.25	0.02	

Abbreviations: Coeff.  =  unstandardized coefficient, SE  =  standard error.

The direct effect of ACEs on spiritual well‐being was −0.21 [−0.35, −0.08]. The indirect effect of ACEs on spiritual well‐being through resilient mindset (effect  =  −0.06, [−0.11, −0.03]), depression (effect  =  −0.14, [−0.22, −0.08]), anxiety (effect  =  −0.09, [−0.17, −0.03]) and stress (effect  =  −0.09, [−0.15, −0.03]) was found to be significant. The results of the serial mediation analysis showed that the indirect effect of ACEs on spiritual well‐being through both resilient mindset and depression (effect  =  −0.03, [−0.04, −0.01]), resilient mindset and anxiety (effect  =  −0.01, [−0.02, −0.01]), and resilient mindset and stress (effect  =  −0.01, [−0.02, −0.01]) was also significant (see Table 3).

**TABLE 3:** Unstandardized total, direct, and indirect effects.

									95%	
Model pathway		Mediator				Outcome	Effect	BootSE	LLCI	ULCI
ACEs	→	Resilient mindset	→			Spiritual well‐being	−0.06	0.02	−0.11	−0.03
ACEs	→	Depression	→			Spiritual well‐being	−0.14	0.04	−0.22	−0.08
ACEs	→	Anxiety	→			Spiritual well‐being	−0.09	0.04	−0.17	−0.03
ACEs	→	Stress	→			Spiritual well‐being	−0.09	0.03	−0.15	−0.03
ACEs	→	Resilient mindset	→	Depression	→	Spiritual well‐being	−0.03	0.01	−0.04	−0.01
ACEs	→	Resilient mindset	→	Anxiety	→	Spiritual well‐being	−0.01	0.01	−0.02	−0.01
ACEs	→	Resilient mindset	→	Stress	→	Spiritual well‐being	−0.01	0.01	−0.02	−0.01
Total indirect effect							−0.43	0.04	−0.52	−0.35
Direct effect							−0.21	0.07	−0.35	−0.08
Total effect							−0.64	0.07	−0.78	−0.51

*Note*: Number of bootstrap samples for percentile bootstrap confidence intervals: 5.000. ACEs were measured on a 0–10 scale and spiritual well‐being on a 0–20 scale; thus, a total effect of −0.64 reflects a 0.64‐point decrease in spiritual well‐being for each one‐unit increase in ACEs.

## 4 Discussion

Spiritual well‐being is considered a dimension of health. It is of great importance to determine the factors and mechanisms which will promote spiritual well‐being. Therefore, this study was to determine the mediating roles of resilient mindset, depression, anxiety, and stress in the link between ACEs and spiritual well‐being, controlling for age and gender. The findings of the current research showed that ACEs had a direct effect on spiritual well‐being. Spiritual well‐being declined in the face of ACEs. Consistent with our results, individuals with a history of sexual/physical abuse felt internal disharmony, purposelessness, and a lack of productivity in their lives, reported being unable to calm themselves down and feeling insecure about their future, which in turn destroyed their spirituality (Sansone et al. 2013). Youth who experienced emotional neglect and emotional abuse in childhood had lower levels of spirituality (Öztekin, Turp, et al. 2025; Prior and Petra 2020). Indeed, spirituality is considered a protective factor for coping with many difficulties (Manning et al. 2019). However, during the period when spirituality is formed, children's adverse experiences appear as a situation that prevents this, and this situation can harm the formation of spiritual well‐being (Shin et al. 2024).

The results of this study revealed that a resilient mindset acted as a mediator in the link between ACEs and spiritual well‐being. Adverse experiences in childhood were associated with lower levels of resilient mindset and, subsequently, lower spiritual well‐being. Previous research has also provided evidence of the mediating role of resilient mindset, which buffered the adverse impact of coronavirus‐related stress on depressive symptoms (Arslan and Coşkun 2024). In addition, individuals exposed to ACEs reported problems with internalizing and externalizing behaviors and lower levels of resilience, which may significantly undermine long‐term biopsychological development (Morgan et al. 2021). Increased resilience was associated with greater spiritual well‐being (Zafari et al. 2023), and ACEs and spirituality influenced subjective well‐being through resilience (Akintunde et al. 2024; Dey et al. 2021). The effects of negative experiences in childhood may continue in later years. A resilient mindset supports people to recover and leap forward—to deal with challenges proactively. This, in turn, contributes to building resilience (Wong et al. 2021). A resilient mindset involves being aware of and understanding individuals’ strengths and enables people to cope with challenges. Thus, cultivating a resilient mindset may have benefits for individuals’ spiritual well‐being.

The study results demonstrated the mediating role of psychological distress in the association between ACEs and spiritual well‐being. Individuals with ACEs reported higher levels of depression, anxiety, and stress, which in turn led to lower spiritual well‐being. Existing literature includes studies demonstrating the mediating role of psychological distress in well‐being. For example, higher social support was associated with less psychological distress, which in turn led to higher satisfaction with life (Dadandi and Çitak 2023). Psychological distress exacerbated the negative impact of anger on individuals' well‐being (Kharwar and Singh 2025). These results reveal that adversities may lead to depression, anxiety and stress, and these have a negative impact on individuals' ability to create meaning or live in harmony with values.

The most salient point of this study was the chain mediating roles of resilient mindset and psychological distress in the link between ACEs and spiritual well‐being. The stronger association between a resilient mindset and depression, compared to anxiety and stress, may reflect differences in the underlying psychological processes linked to ACEs. Depressive symptoms are more closely related to enduring negative cognitive schemas, hopelessness, and meaning disruption, which are directly targeted by resilient cognitive appraisals. In contrast, anxiety and stress are more sensitive to situational uncertainty and contextual demands, potentially limiting the influence of a resilient mindset on these outcomes. In addition, ACEs can cause mental health problems and hinder spiritual well‐being (Hinojosa and Hinojosa 2024; Shin et al. 2024). A resilient mindset is a resource that allows people to cope with adversity, and even if those with this resource experienced difficult circumstances in their childhood, they can maintain a positive outlook and perceive these adversities as opportunities for growth. However, those who have not developed a resilient mindset may experience mental health problems such as depression, anxiety, and stress, which may adversely impact their well‐being (Arslan and Wong 2024). We can conclude that a resilient mindset has a protective effect against the deleterious effects of ACEs on spiritual well‐being, while depression, anxiety, and stress have an exacerbating effect on this relationship. However, it should be remembered that it is equally possible that current spiritual struggles or depression influence the recall of childhood experiences or the self‐assessment of one's resilient mindset.

Chain mediation findings suggest that the impact of ACEs on spiritual well‐being progresses gradually through cognitive and emotional processes. Individuals exposed to ACEs may develop a less resilient mindset, weakening their assessments of their ability to cope with stressful life events and leading them to perceive themselves as ineffective and out of control (Lazarus 1984). This cognitive vulnerability, combined with negative core schemas shaped by early adverse experiences, increases the likelihood of developing symptoms of psychological distress (Beck 1967). Increased psychological distress acts as a mechanism that weakens spiritual well‐being by suppressing an individual's capacity to evaluate life events within a meaningful whole and their sense of purpose (Park 2010; Sorajjakool et al. 2008).

These findings suggest that interventions aimed at supporting spiritual well‐being should not focus solely on symptom reduction but rather on enhancing an individual's capacity to make sense of life events, their sense of consistency with their value system, and their ability to develop meaningful coping mechanisms in the face of existential threats. Meaning‐based approaches can strengthen the fundamental components of spiritual well‐being by supporting the integration of negative childhood experiences into an individual's life story and the creation of meaning in the face of uncontrollable experiences (Wong 2013). Existential coping processes, by involving acceptance, purpose redefinition, and the creation of a broader life context instead of avoidance in the face of pain, uncertainty, and loss, function as an important mechanism that reduces the suppressive effect of psychological distress on spiritual well‐being (Pargament 2011; Yalom 1980). From a developmental psychopathology perspective, ACEs affect the early shaping of an individual's existential assumptions about the world, self, and the meaning of life (Cicchetti and Toth 2009; Kwok et al. 2024). In this regard, a resilient mindset is positioned not only as a cognitive coping mechanism but also as a developmental existential resource that supports an individual's capacity to make life meaningful in the face of pain and uncertainty. Since childhood, adolescence, and emerging adulthood are periods of restructuring of identity, values, and meaning systems, and when cognitive flexibility is relatively high, interventions targeting resilient mindsets and meaning‐making during these periods can be argued to play a critical protective role in terms of long‐term spiritual well‐being and psychological adjustment (Arnett 2000; Vanistendael 2007). On the other hand, psychological distress, as an outcome of adverse experiences, along with existential problems, can be considered a risk factor for spiritual well‐being (Hou et al. 2022; Yildirim Kurtulus et al. 2025).

The present study offers theoretical and practical implications. This study revealed that individuals who experienced adversities in childhood have difficulty in developing a resilient mindset, which increases the probability of experiencing depression, anxiety, and stress, and thus, these reduce enhanced spiritual well‐being. Therefore, practices can be developed to help individuals develop their resilient mindset skills and relieve their psychological distress. More importantly, awareness and prevention programs can be organized for parents or caregivers about the future benefits of a healthy environment in which children grow up, the importance of a healthy child‐parent relationship, and the negative effects of neglect and abuse. Parents should be informed that creating safe and healthy environments that allow children to play, explore, and maximize their capacities facilitates the development of protective factors such as a resilient mindset.

The study results should be considered in the context of some limitations. The sample consists exclusively of Turkish university students, with a significant gender imbalance (75.4% female). This limits the generalizability of the findings to other populations, such as non‐student adults, individuals from different cultural or socioeconomic backgrounds, clinical populations, or more gender‐balanced groups. The experiences and coping mechanisms of university students may not represent those of the wider population affected by ACEs. Future studies should replicate this study, paying attention to gender distribution and focusing on diverse populations. The use of convenience sampling via online surveys may lead to selection bias. This could limit the generalizability of these findings. Future studies should be conducted using random sampling. The data were self‐reported in a single session. Relying on self‐reported measures may carry a risk of source bias and common‐method variance. Researchers should incorporate multi‐method approaches. This study is cross‐sectional, which precludes causal inference. Researchers should plan longitudinal and experimental studies. Although age and gender were statistically controlled in the current study, other contextual factors, such as religiosity and social support, can be the focus of future studies. Since the Resilient Mindset Scale has recently been developed in Turkish culture, researchers should focus on studies related to this concept in different age groups. The reliability of the Spiritual Well‐being Scale was moderate in this sample (*α* = 0.72). Researchers should retest the reliability of the scale on different samples with similar characteristics.

In conclusion, spiritual well‐being is a crucial component of overall health. Therefore, promoting spiritual well‐being is a priority. This study revealed the negative association between ACEs and spiritual well‐being. Resilient mindset, depression, anxiety, and stress were psychological variables that mediated this relationship. These results show that experiencing adversities in childhood prevents individuals from developing the ability to cope with a negative situation, and the lack of these strengths makes individuals vulnerable to depression, anxiety, and stress, and as a result, their spiritual well‐being decreases. This study highlights the importance of developing a resilient mindset and reducing levels of psychological distress to mitigate the negative impact of ACEs on spiritual well‐being. These findings suggest that psychological counseling and psychoeducation programs in university settings should utilize the assessments of spiritual well‐being, a resilient mindset or psychological distress in conjunction with brief ACEs screening, and that at‐risk students should be identified and intervention programs organized early.

## Author Contributions

**Gülçin Güler Öztekin**: conceptualization, data collection, data preparation, data analysis, writing – original draft. **Murat Yıldırım**: supervision, validation, and writing – review and editing. **Nouf Abdullah Alshehri**: writing – review and editing. **Abdulmohsen Mohammed Abdullah Alkhulayfi**: writing – review and editing.

## Funding

The authors have nothing to report.

## Ethics Statement

This study was approved by the Institutional Review Board of the Agri Ibrahim Cecen University in accordance with the Helsinki Declaration 2013 (Ethic Code: E‐95531838‐050.99‐124706). This is a statement to confirm that all methods were carried out in accordance with relevant guidelines and regulations. The researcher considered the ethics of conducting research throughout the research process. Before the data collection, all participants were informed of the research objectives, data collection method, and the right of acceptance to accept or refuse to participate in the research. Consent to participate in the study was provided via the first page of the online survey.

## Consent

Consent was obtained from all participants included in the study.

## Conflicts of Interest

The authors declare no conflicts of interest.

## Data Availability

The data supporting this study's findings are available from the corresponding author upon reasonable request.

## References

[brb371227-bib-0001] Akintunde, T. Y. , S. O. Isangha, A. O. Iwuagwu , and A. Adedeji . 2024. “Adverse Childhood Experiences and Subjective Well‐Being of Migrants: Exploring the Role of Resilience and Gender Differences.” Global Social Welfare 11, no. 3: 243–255. 10.1007/s40609-023-00310-w.

[brb371227-bib-0002] Arnett, J. J. 2000. “Emerging Adulthood: A Theory of Development From the Late Teens Through the Twenties.” American Psychologist 55, no. 5: 469. 10842426

[brb371227-bib-0003] Arslan, G. 2025. “Childhood Maltreatment, Spiritual Wellbeing, and Stress‐Related Growth in Emerging Adults: A Conditional Approach to Responsibility.” Current Psychology 44: 1372–1381. 10.1007/s12144-025-07280-6.

[brb371227-bib-0004] Arslan, G. , and M. Coşkun . 2024. “Coronavirus–Related Stressors, Resilient Mindset, Loneliness, Depressive Symptoms in College Students: Testing a Moderated Mediation Model.” Psychological Reports 127, no. 4: 1633–1651. 10.1177/00332941221139721. 36377653

[brb371227-bib-0005] Arslan, G. , and P. Wong . 2024. “Embracing Life's Challenges: Developing a Tool for Assessing Resilient Mindset in Second Wave Positive Psychology.” Journal of Happiness and Health 4, no. 1: 1–10. 10.47602/johah.v4i1.53.

[brb371227-bib-0006] Arslan, G. , and M. Yıldırım . 2021. “Meaning‐Based Coping and Spirituality During the COVID‐19 Pandemic: Mediating Effects on Subjective Well‐Being.” Frontiers in Psychology 12: 646572. 10.3389/fpsyg.2021.646572. 33935906 PMC8082073

[brb371227-bib-0007] Beck, A. T. 1967. Depression: Clinical, Experimental, and Theoretical Aspects. Haper & Row.

[brb371227-bib-0008] Bhutta, Z. A. , S. Bhavnani, T. S. Betancourt, M. Tomlinson,, and V. Patel . 2023. “Adverse Childhood Experiences and Lifelong Health.” Nature Medicine 29, no. 7: 1639–1648. 10.1038/s41591-023-02426-0. 37464047

[brb371227-bib-0009] Boullier, M. , and M. Blair . 2018. “Adverse Childhood Experiences.” Paediatrics and Child Health 28, no. 3: 132–137. 10.1016/j.paed.2017.12.008.

[brb371227-bib-0010] Bredle, J. M. , J. M. Salsman, S. M. Debb, B. J. Arnold,, and D. Cella . 2011. “Spiritual Well‐Being as a Component of Health‐Related Quality of Life: the Functional Assessment of Chronic Illness Therapy—Spiritual Well‐Being Scale (FACIT‐Sp).” Religions 2, no. 1: 77–94. 10.3390/rel2010077.

[brb371227-bib-0011] Chaiyasit, Y. , N. Kunakote, P. Kotta, K. Chanbunlawat,, and P. Piboonrungroj . 2020. “Predicting Factors of Spiritual Well‐Being Among People Living With HIV/AIDS.” Bangkok Medical Journal 16, no. 1: 26–26. 10.31524/bkkmedj.2020.11.006.

[brb371227-bib-0012] Chirico, F. , K. Batra, R. Batra, et al. 2023. “Spiritual Well‐Being and Burnout Syndrome in Healthcare: A Systematic Review.” Journal of Health and Social Science 8, no. 1: 13–32.

[brb371227-bib-0013] Cicchetti, D. 2013. “Annual Research Review: Resilient Functioning in Maltreated Children–Past, Present, and Future Perspectives.” Journal of Child Psychology and Psychiatry 54, no. 4: 402–422. 10.1111/j.1469-7610.2012.02608.x. 22928717 PMC3514621

[brb371227-bib-0014] Cicchetti, D. , and R. Rizley . 1981. “Developmental Perspectives on the Etiology, Intergenerational Transmission, and Sequelae of Child Maltreatment.” New Directions for Child and Adolescent Development 1981, no. 11: 31–55. 10.1002/cd.23219811104.

[brb371227-bib-0015] Cicchetti, D. , and S. L. Toth . 2009. “The Past Achievements and Future Promises of Developmental Psychopathology: The Coming of Age of a Discipline.” Journal of Child Psychology and Psychiatry 50, no. 1‐2: 16–25. 10.1111/j.1469-7610.2008.01979.x. 19175810 PMC3531893

[brb371227-bib-0016] Coppola, I. , N. Rania, R. Parisi,, and F. Lagomarsino . 2021. “Spiritual Well‐Being and Mental Health During the COVID‐19 Pandemic in Italy.” Frontiers in Psychiatry 12: 626944. 10.3389/fpsyt.2021.626944. 33868047 PMC8046904

[brb371227-bib-0017] Curran, P. J. , S. G. West,, and J. F. Finch . 1996. “The Robustness of Test Statistics to Nonnormality and Specification Error in Confirmatory Factor Analysis.” Psychological Methods 1, no. 1: 16. 10.1037/1082-989X.1.1.16.

[brb371227-bib-0018] Dadandi, I. , and S. Çitak . 2023. “Psychological Distress Mediates the Relationship between Social Support and Satisfaction With Life.” International Journal of Contemporary Educational Research 10, no. 3: 724–734. 10.52380/ijcer.2023.10.3.543.

[brb371227-bib-0019] Dey, N. E. Y. , B. Amponsah,, and C. B. Wiafe‐Akenteng . 2021. “Spirituality and Subjective Well‐Being of Ghanaian Parents of Children With Special Needs: The Mediating Role of Resilience.” Journal of Health Psychology 26, no. 9: 1377–1388. 10.1177/1359105319873956. 31516014

[brb371227-bib-0020] Fabbris, J. L. , A. C. Mesquita, S. Caldeira, A. M. P. Carvalho,, and E. C. D. Carvalho . 2017. “Anxiety and Spiritual Well‐Being in Nursing Students: A Cross‐Sectional Study.” Journal of Holistic Nursing 35, no. 3: 261–270. 10.1177/0898010116655004. 27324738

[brb371227-bib-0021] Fossion, P. , C. Leys, C. Kempenaers, S. Braun, P. Verbanck,, and P. Linkowski . 2015. “Beware of Multiple Traumas in PTSD Assessment: The Role of Reactivation Mechanism in Intrusive and Hyper‐Arousal Symptoms.” Aging & Mental Health 19, no. 3: 258–263. 10.1080/13607863.2014.924901. 24927132

[brb371227-bib-0022] Gomez, R. , and J. W. Fisher . 2003. “Domains of Spiritual Well‐Being and Development and Validation of the Spiritual Well‐Being Questionnaire.” Personality and Individual Differences 35, no. 8: 1975–1991. 10.1016/S0191-8869(03)00045-X.

[brb371227-bib-0023] Gündüz, A. , A. B. Yaşar, İ. Gündoğmuş , C. Savran , and E. Konuk . 2018. “Çocukluk Çağı Olumsuz yaşantılar Ölçeği Türkçe Formunun Geçerlilik Ve Güvenilirlik Çalışması.” Anatolian Journal of Psychiatry 19, no. 1: 68–75. 10.5455/apd.294158.

[brb371227-bib-0024] Hansen, J. , C. Hartwell,, and S. R. Madsen . 2021. “The Impact of COVID‐19 on Utah Women and Work: Resilient Mindset and Wellbeing.” Research and Policy Brief 36: 1.

[brb371227-bib-0025] Hatun, O. 2024. “Investigating the Mediating Roles of Hopelessness and Psychological Distress in the Relationship Between Psychological Vulnerability and Well‐Being Among Married Individuals.” Spiritual Psychology and Counseling 9, no. 3: 283–300. 10.37898/spiritualpc.1505961.

[brb371227-bib-0026] Hawes, D. J. , and J. L. Allen . 2023. “A Developmental Psychopathology Perspective on Adverse Childhood Experiences (ACEs): Introduction to the Special Issue.” Research on Child and Adolescent Psychopathology 51, no. 12: 1715–1723. 10.1007/s10802-023-01100-w. 37421507 PMC10661772

[brb371227-bib-0027] Hedrick, J. , V. Bennett, J. Carpenter , et al. 2021. “A Descriptive Study of Adverse Childhood Experiences and Depression, Anxiety, and Stress Among Undergraduate Nursing Students.” Journal of Professional Nursing 37, no. 2: 291–297. 10.1016/j.profnurs.2021.01.007. 33867083

[brb371227-bib-0028] Henry, J. D. , and J. R. Crawford . 2005. “The Short‐Form Version of the Depression Anxiety Stress Scales (DASS‐21): Construct Validity and Normative Data in a Large Non‐Clinical Sample.” British Journal of Clinical Psychology 44, no. 2: 227–239. 10.1348/014466505x29657. 16004657

[brb371227-bib-0029] Hinojosa, M. S. , and R. Hinojosa . 2024. “Positive and Adverse Childhood Experiences and Mental Health Outcomes of Children.” Child Abuse & Neglect 149: 106603. 10.1016/j.chiabu.2023.106603. 38141478

[brb371227-bib-0030] Hoskeri, A. G. 2024. Master Resilient Mindset: Thrive through Adversity, Transform Challenges Into Opportunities, Build Your Inner Strength, and Be Unstoppable. Findaway Voices.

[brb371227-bib-0031] Hou, H. , C. Zhang, J. Tang , et al. 2022. “Childhood Experiences and Psychological Distress: Can Benevolent Childhood Experiences Counteract the Negative Effects of Adverse Childhood Experiences?” Frontiers in Psychology 13: 800871. 10.3389/fpsyg.2022.800871. 35282200 PMC8914177

[brb371227-bib-0032] Janů, A. , K. Malinakova, A. Kosarkova , and P. Tavel . 2022. “Associations of Childhood Trauma Experiences With Religious and Spiritual Struggles.” Journal of Health Psychology 27, no. 2: 292–304. 10.1177/1359105320950793. 32830557

[brb371227-bib-0033] Kalmakis, K. A. , and G. E. Chandler . 2015. “Health Consequences of Adverse Childhood Experiences: A Systematic Review.” Journal of the American Association of Nurse Practitioners 27, no. 8: 457–465. 10.1002/2327-6924.12215. 25755161

[brb371227-bib-0034] Kharwar, S. , and P. Singh . 2025. “Direct and Indirect Effect of Anger and Cognitive Failures on Subjective Well‐Being: Mediating Role of Psychological Distress.” Psychological Studies 70: 1–16. 10.1007/s12646-025-00824-7.

[brb371227-bib-0035] Kutner, M. J. , C. J. Nachtsheim, J. Neter , and W. Li . 2004. Applied Linear Statistical Models, 5th ed. McGraw‐Hill.

[brb371227-bib-0036] Kwok, S. Y. , J. Jiang,, and S. Fang . 2024. “Presence of Meaning in Life and Meaning Confusion Mediate the Effects of Adverse Childhood Experiences on Mental Health Among University Students.” Applied Psychology: Health and Well‐Being 16, no. 1: 179–197. 10.1111/aphw.12478. 37524657

[brb371227-bib-0037] Lazarus, R. S. 1984. Stress, Appraisal, and Coping 445. Springer.

[brb371227-bib-0038] Liu, S. , A. Lithopoulos, C.‐Q. Zhang, M. A. Garcia‐Barrera,, and R. E. Rhodes . 2021. “Personality and Perceived Stress During COVID‐19 Pandemic: Testing the Mediating Role of Perceived Threat and Efficacy.” Personality and Individual Differences 168: 110351. 10.1016/j.paid.2020.110351. 32863508 PMC7442020

[brb371227-bib-0039] Lovibond, P. F. , and S. H. Lovibond . 1995. “The Structure of Negative Emotional States: Comparison of the Depression Anxiety Stress Scales (DASS) With the Beck Depression and Anxiety Inventories.” Behaviour Research and Therapy 33, no. 3: 335–343. 10.1016/0005-7967(94)00075-U. 7726811

[brb371227-bib-0040] Makkaoui, M. , F. Z. Hannoun, K. Ouazizi , et al. 2024. “Spirituality as Predictor of Psychological Well‐Being at Work in the Moroccan Context: A Cross‐Sectional Study.” Journal of Health and Social Science 9, no. 2: 279–293.

[brb371227-bib-0041] Manning, L. , M. Ferris, C. Narvaez Rosario, M. Prues,, and L. Bouchard . 2019. “Spiritual Resilience: Understanding the Protection and Promotion of Well‐Being in the Later Life.” Journal of Religion, Spirituality & Aging 31, no. 2: 168–186. 10.1080/15528030.2018.1532859. PMC7743140 33335455

[brb371227-bib-0042] Manyema, M. , S. A. Norris,, and L. M. Richter . 2018. “Stress Begets Stress: The Association of Adverse Childhood Experiences With Psychological Distress in the Presence of Adult Life Stress.” BMC Public Health [Electronic Resource] 18: 1–12. 10.1186/s12889-018-5767-0. PMC6034311 29976168

[brb371227-bib-0043] Maral, S. , H. Bilmez,, and S. A. Satici . 2024. “Positive Childhood Experiences and Spiritual Well‐Being: Psychological Flexibility and Meaning‐Based Coping as Mediators in Turkish Sample.” Journal of Religion and Health 63, no. 4: 2709–2726. 10.1007/s10943-024-02079-4. 38913254 PMC11319421

[brb371227-bib-0044] McCormick, W. H. , T. D. Carroll, B. M. Sims,, and J. Currier . 2017. “Adverse Childhood Experiences, Religious/Spiritual Struggles, and Mental Health Symptoms: Examination of Mediation Models.” Mental Health, Religion & Culture 20, no. 10: 1042–1054. 10.1080/13674676.2018.1440544.

[brb371227-bib-0045] Morgan, C. A. , Y.‐H. Chang, O. Choy, M.‐C. Tsai,, and S. Hsieh . 2021. “Adverse Childhood Experiences Are Associated With Reduced Psychological Resilience in Youth: A Systematic Review and Meta‐Analysis.” Children 9, no. 1: 27. 10.3390/children9010027. 35053652 PMC8773896

[brb371227-bib-0046] Moustafa, A. A. , J. J. Crouse, M. M. Herzallah, et al. 2020. “Depression Following Major Life Transitions in Women: A Review and Theory.” Psychological Reports 123, no. 5: 1501–1517. 10.1177/0033294119872209. 31470771

[brb371227-bib-0047] Nahardani, S. Z. , F. Ahmadi, S. Bigdeli,, and K. Soltani Arabshahi . 2019. “Spirituality in Medical Education: A Concept Analysis.” Medicine, Health Care and Philosophy 22: 179–189. 10.1007/s11019-018-9867-5. 30206758

[brb371227-bib-0048] Özdemir, A. A. , F. Kavak Buda, G. Dural,, and A. Gültekin . 2023. “The Relationship Between Spiritual Well‐Being, Life Satisfaction and Hope in Elderly Individuals in Turkey.” Journal of Religion and Health 62, no. 5: 3123–3136. 10.1007/s10943-022-01517-5. 35122554

[brb371227-bib-0049] Öztekin, G. G. , J. Gómez‐Salgado,, and M. Yıldırım . 2025. “Future Anxiety, Depression and Stress Among Undergraduate Students: Psychological Flexibility and Emotion Regulation as Mediators.” Frontiers in Psychology 16: 1517441. 10.3389/fpsyg.2025.1517441. 39958768 PMC11825509

[brb371227-bib-0050] Öztekin, G. G. , H. H. Turp, N. A. Alzahrani, J. Gómez‐Salgado,, and M. Yıldırım . 2025. “Understanding the Associations Between Adverse Childhood Experiences and Spiritual Well‐Being Among Turkish University Students: Testing the Mediating Roles of Rumination and Forgiveness.” Brain and Behavior 15, no. 10: e70923. 10.1002/brb3.70923. 41116641 PMC12537836

[brb371227-bib-0051] Pargament, K. I. 2011. Spiritually Integrated Psychotherapy: Understanding and Addressing the Sacred. Guilford Press.

[brb371227-bib-0052] Park, C. L. 2010. “Making Sense of the Meaning Literature: An Integrative Review of Meaning Making and Its Effects on Adjustment to Stressful Life Events.” Psychological Bulletin 136, no. 2: 257. 20192563 10.1037/a0018301

[brb371227-bib-0053] Prior, M. K. , and M. Petra . 2020. “Assessing the Effects of Childhood Multitype Maltreatment on Adult Spirituality.” Journal of Child & Adolescent Trauma 13: 469–480. 10.1007/s40653-019-00288-8. 33269046 PMC7683659

[brb371227-bib-0054] Rizzo, A. , M. Yıldırım, I. A. Aziz, et al. 2023. “Anxiety and Coping Strategies Among Italian‐Speaking Physicians: a Comparative Analysis of the Contractually Obligated and Voluntary Care of COVID‐19 Patients.” Healthcare 11, no. 23: 3044. 38063612 10.3390/healthcare11233044 PMC10706344

[brb371227-bib-0055] Roussis, P. , and A. Wells . 2008. “Psychological Factors Predicting Stress Symptoms: Metacognition, Thought Control, and Varieties of Worry.” Anxiety, Stress, & Coping 21, no. 3: 213–225. 10.1080/10615800801889600. 18938290

[brb371227-bib-0056] Sansone, R. A. , A. R. Kelley,, and J. S. Forbis . 2013. “Abuse in Childhood and Religious/Spiritual Status in Adulthood Among Internal Medicine Outpatients.” Journal of Religion and Health 52: 1085–1092. 10.1007/s10943-012-9582-0. 22395752

[brb371227-bib-0057] Shin, H. K. , X. Tong, W. Turner,, and J. S. Lyons . 2024. “Examining the Impact of Spirituality Religious Strength on Behavioral and Emotional Health Among Youth With Multiple Adverse Childhood Experiences.” Children and Youth Services Review 164: 107847. 10.1016/j.childyouth.2024.107847.

[brb371227-bib-0058] Shin, S. H. , S. Hassamal,, and L. P. Groves . 2015. “Examining the Role of Psychological Distress in Linking Childhood Maltreatment and Alcohol Use in Young Adulthood.” American Journal on Addictions 24, no. 7: 628–636. 10.1111/ajad.12276. 26346173 PMC5749918

[brb371227-bib-0059] Sorajjakool, S. , V. Aja, B. Chilson, J. Ramírez‐Johnson,, and A. Earll . 2008. “Disconnection, Depression, and Spirituality: A Study of the Role of Spirituality and Meaning in the Lives of Individuals With Severe Depression.” Pastoral Psychology 56, no. 5: 521–532. 10.1007/s11089-008-0125-2.

[brb371227-bib-0060] Sroufe, L. A. 1997. “Psychopathology as an Outcome of Development.” Development and Psychopathology 9, no. 2: 251–268. 10.1017/S0954579497002046. 9201444

[brb371227-bib-0061] Strine, T. W. , V. J. Edwards, S. R. Dube, et al. 2012. “The Mediating Sex‐Specific Effect of Psychological Distress on the Relationship Between Adverse Childhood Experiences and Current Smoking Among Adults.” Substance Abuse Treatment, Prevention, and Policy 7: 1–13. 10.1186/1747-597X-7-30. 22788356 PMC3541176

[brb371227-bib-0062] Vanistendael, S. 2007. “Resilience and Spirituality.” In Resilience in Palliative Care: Achievement in Adversity, 115–135. Oxford University Press.

[brb371227-bib-0063] Vingerhoets, A. 2008. “The Assessment of Stress.” In Probing Experience: from Assessment of User Emotions and Behaviour to Development of Products, 109–117. Springer.

[brb371227-bib-0064] Wong, P. T. 2013. “Toward a Dual‐Systems Model of What Makes Life Worth living.” In The Human Quest for Meaning, 3–22. Routledge.

[brb371227-bib-0065] Wong, P. T. 2020. Made for Resilience and Happiness: Effective Coping With COVID‐19 According to Viktor E. Frankl and Paul TP Wong. INPM Press.

[brb371227-bib-0066] Wong, P. T. , C.‐H. Mayer,, and G. Arslan . 2021. “COVID‐19 and Existential Positive Psychology (PP2. 0): The New Science of Self‐Transcendence.” Frontiers in Psychology 12: 800308. 10.3389/fpsyg.2021.800308. 34956025 PMC8699172

[brb371227-bib-0067] Yalom, I. 1980. Existential Psychotherapy Basic Books. Library of Congress Cataloging in Publication Data,.

[brb371227-bib-0068] Yıldırım, M. , H. Batmaz, H. Yıldırım‐Kurtuluş,, and E. Kurtuluş . 2025. “Associations Between Psychological Capital, Internalizing and Externalizing Problems, Perceived Stress, Emotional, Social, and Psychological Well‐Being in Adolescents.” Youth & Society 57: 878–902.

[brb371227-bib-0069] Yıldırım, M. , S. Cengiz, I. A. Aziz, A. Ziapour,, and M. E. Turan . 2024a. “Posttraumatic Stress Disorder (PTSD), Psychological Flexibility and Psychological Adjustment Problems: Turkish Validation of the PTSD Checklist for Short Form DSM‐5 (PCL‐5‐S).” European Journal of Trauma & Dissociation 8, no. 1: 100381.

[brb371227-bib-0070] Yıldırım, M. , Ü. Dilekçi, F. Marcatto,, and J. Gómez‐Salgado . 2024b. “Validation of a Turkish Translation of the Perceived Occupational Stress Scale and Measurement Invariance Across Turkish and Italian workers.” In Risk Management and Healthcare Policy, 261–268. Dove Medical Press Limited. 10.2147/RMHP.S437312 PMC10838499 38313396

[brb371227-bib-0071] Yıldırım, M. , and A. Özaslan . 2022. “Worry, Severity, Controllability, and Preventive Behaviours of COVID‐19 and Their Associations With Mental Health of Turkish Healthcare Workers Working at a Pandemic Hospital.” International Journal of Mental Health and Addiction 20, no. 4: 2306–2320. 33686345 10.1007/s11469-021-00515-0 PMC7928196

[brb371227-bib-0072] Yildirim Kurtulus, H. , S. A. Satici,, and M. E. Deniz . 2025. “Adverse Childhood Experiences and Spiritual Well‐Being Among Turkish People: A Serial Mediation Through Meaningful Living and Death Obsession.” Journal for the Study of Spirituality 1: 1–17. 10.1080/20440243.2025.2558730.

[brb371227-bib-0073] Yilmaz, F. B. , and S. A. Satici . 2024. “Childhood Maltreatment and Spiritual Well‐Being: Intolerance of Uncertainty and Emotion Regulation as Mediators in Turkish Sample.” Journal of Religion and Health 63, no. 3: 2380–2396. 10.1007/s10943-023-01965-7. 38070045

[brb371227-bib-0074] Yılmaz, Ö. , H. Boz,, and A. Arslan . 2017. “Depresyon Anksiyete Stres Ölçeğinin (DASS 21) Türkçe Kisa Formunun Geçerlilik‐güvenilirlik çalişmasi.” Finans Ekonomi ve Sosyal Araştırmalar Dergisi 2, no. 2: 78–91.

[brb371227-bib-0075] Zafari, M. , M. Sadeghipour Roudsari, S. Yarmohammadi, A. Jahangirimehr,, and T. Marashi . 2023. “Investigating the Relationship Between Spiritual Well‐Being, Resilience, and Depression: A Cross‐Sectional Study of the Elderly.” Psychogeriatrics 23, no. 3: 442–449. 10.1111/psyg.12952. 36892004

[brb371227-bib-0076] Zeidner, M. , and G. Matthews . 2010. Anxiety 101. Springer Publishing Company.

[brb371227-bib-0077] Zhu, Y. , G. Zhang, S. Zhan,, and T. Anme . 2024. “Longitudinal Effects of Parental Adverse Childhood Experiences on Offspring Problematic media Use: The Serial Mediating Role of Psychological Distress and Harsh Discipline.” Child Abuse & Neglect 155: 106965. 10.1016/j.chiabu.2024.106965. 39106783

[brb371227-bib-0078] Zinnbauer, B. J. , K. I. Pargament,, and A. B. Scott . 1999. “The Emerging Meanings of Religiousness and Spirituality: Problems and Prospects.” Journal of Personality 67, no. 6: 889–919. 10.1111/1467-6494.00077.

